# Constructing Rigorous and Broad Biosurveillance Networks for Detecting Emerging Zoonotic Outbreaks

**DOI:** 10.1371/journal.pone.0124037

**Published:** 2015-05-06

**Authors:** Mac Brown, Leslie Moore, Benjamin McMahon, Dennis Powell, Montiago LaBute, James M. Hyman, Ariel Rivas, Mark Jankowski, Joel Berendzen, Jason Loeppky, Carrie Manore, Jeanne Fair

**Affiliations:** 1 University of California-Santa Barbara, Department of Economics, Santa Barbara, California, 93111, United States of America; 2 Statistical Sciences, Los Alamos National Laboratory, Los Alamos, New Mexico, 87545, United States of America; 3 Los Alamos National Laboratory, Theoretical Biology and Biophysics, Los Alamos, New Mexico, 87545, United States of America; 4 Energy and Infrastructure Analysis, Los Alamos National Laboratory, Los Alamos, New Mexico, 87545, United States of America; 5 Lawrence Livermore National Laboratory, Applied Statistics Group—Computational Engineering Division, Mailstop L-174, 7000 East Ave. Livermore, California, 94550, United States of America; 6 Department of Mathematics, Tulane University, New Orleans, Louisiana, 70118, United States of America; 7 Center for Global Health, Health Sciences Center, University of New Mexico, Albuquerque, New Mexico, 87131, United States of America; 8 Minnesota Pollution Control Agency, Environmental Analysis & Outcomes Division, St. Paul, Minnesota, 55155, United States of America; 9 Los Alamos National Laboratory, Applied Modern Physics, Mailstop D454, Los Alamos, New Mexico, 87545, United States of America; 10 University of British Columbia, Okanagan, 3333 University Way, Kelowna, B.C. V1V 1V7, Canada; 11 Center for Computational Science, Tulane University, New Orleans, Louisiana, 70118, United States of America; 12 Los Alamos National Laboratory, Environmental Stewardship, K404, Los Alamos, New Mexico, 87545, United States of America; National Institutes of Health, UNITED STATES

## Abstract

Determining optimal surveillance networks for an emerging pathogen is difficult since it is not known beforehand what the characteristics of a pathogen will be or where it will emerge. The resources for surveillance of infectious diseases in animals and wildlife are often limited and mathematical modeling can play a supporting role in examining a wide range of scenarios of pathogen spread. We demonstrate how a hierarchy of mathematical and statistical tools can be used in surveillance planning help guide successful surveillance and mitigation policies for a wide range of zoonotic pathogens. The model forecasts can help clarify the complexities of potential scenarios, and optimize biosurveillance programs for rapidly detecting infectious diseases. Using the highly pathogenic zoonotic H5N1 avian influenza 2006-2007 epidemic in Nigeria as an example, we determined the risk for infection for localized areas in an outbreak and designed biosurveillance stations that are effective for different pathogen strains and a range of possible outbreak locations. We created a general multi-scale, multi-host stochastic SEIR epidemiological network model, with both short and long-range movement, to simulate the spread of an infectious disease through Nigerian human, poultry, backyard duck, and wild bird populations. We chose parameter ranges specific to avian influenza (but not to a particular strain) and used a Latin hypercube sample experimental design to investigate epidemic predictions in a thousand simulations. We ranked the risk of local regions by the number of times they became infected in the ensemble of simulations. These spatial statistics were then complied into a potential risk map of infection. Finally, we validated the results with a known outbreak, using spatial analysis of all the simulation runs to show the progression matched closely with the observed location of the farms infected in the 2006-2007 epidemic.

## Introduction

Disease surveillance is an difficult challenge on both global and local scales. Surveillance planning can improve the effectiveness and ability to detect a disease during and prior to epidemic. The high costs of surveillance limits the number of locations where the it can be placed. Epidemiological modeling and uncertainty analysis can help optimize these locations to maximize the probability of detecting an emerging epidemic. We develop a methodological approach to assist in surveillance planning by identifying potential disease hotspots. The locations are identified from a wide range of forecasts from a multi-scale, stochastic, geo-spatial epidemiological model that includes agricultural animals, wildlife, and humans.

Emerging infections have an enormous impact on animal and public health, food supply, and local/regional economies. In particular, there continues to be concern surrounding newly emerging strains of influenzas such as the H1N1pandemic of 2009; the highly pathogenic avian influenzas H5N1 that has caused outbreaks since 1997 across Asia, Europe, and Africa [1,2]; and H7N9 which emerged in the spring of 2013 in China [3]. Infectious disease outbreaks, such as highly pathogenic avian influenzas that spread to agricultural animals, can be costly [4,5]. The high cost includes both the direct mortality of animals from infections and the depopulation culling policies designed to control the spread of pathogens and protect the safety of international trade. Zoonotic pathogens are pathogens in nonhuman vertebrate animals that may be transmitted to humans under natural conditions. Early detection require integrated surveillance in animals and humans for both predicting and reducing the spread of these infections [6].

Pathogen surveillance in animals is usually the responsibility of government departments of agriculture. Its quality varies greatly among countries and typically does not include wildlife. The recently restructured Animal Health Information System of the World Organization for Animal Health (OIE) includes an International Monitoring System, and an International Early Warning System through which member countries have agreed to report immediately any of six categories of animal disease occurrences[7]. All of the OIE reportable pathogens, including many important zoonoses, affect international trade, and their early detection is of joint interest to the international community.

Even with the OIE monitoring and reporting systems, there is a large amount of uncertainty on how to best begin and plan for surveillance within a region. The 2011 United States Government Accountability Office report [8] notes that new disease-reporting systems could help professionals recognize unusual disease signals and analyze their meaning. Most planning for biosurveillance occurs after an outbreak has been detected. This creates inherent limitations that affect the speed with which their results can be determined, communicated, and acted upon.

The term biosurveillance is defined as the process of gathering and combining information with appropriate analysis and interpretation that might relate to disease activity and threats to human or animal health—whether infectious, toxic, metabolic, or otherwise, and regardless of intentional or natural origin—in order to achieve early warning of health threats, early detection of health events, and overall situational awareness of disease activity. Over time, four types of surveillance have been applied to monitoring diseases: active, passive, sentinel, and syndromic. Biosurveillance of zoonotic diseases combines the inherent complexity of the pathogen with uncertainties of how humans and agricultural animals behave and interact, as well as the ecology of wildlife, vectors, and environmental changes.

Wildlife biosurveillance could be enhanced by developing three complementary approaches to overcome the specific constraints associated with infectious disease surveillance: monitoring based on a risk analysis, monitoring of sentinel animals, and syndromic surveillance [9]. Biosurveillance based on risk analysis can help guide the surveillance networks prior to outbreaks to increase the effectiveness of the detecting the disease in new areas and reduce the time between detection and implementation of mitigations.

Highly pathogenic avian influenza H5N1was first detected in Africa in Kaduna State of Nigeria in February 2006 [10]. This was followed by other Nigerian states reporting H5N1 virus infections among millions of domestic and wild birds, with the last recorded outbreak occurring in northern Nigeria in late 2008. The single human case and death due to infection with H5N1 virus occurred in February 2007 [11]. Serological evidence show little evidence of transmission of avian influenza to agricultural farmers (2.4%) [12] and poultry workers (0%) [13].

Highly pathogenic H5N1 influenza in Nigeria is an example of an emerging zoonotic disease outbreak of high human health importance, with equally high economic costs, infecting all three primary types of hosts—humans, agricultural animals, and wildlife. The Nigerian epidemic can be used to validate epidemiological models and apply sensitivity, uncertainty, and risk analyses to investigate their applicability to biosurveillance planning. The validation process must account for both spatial and temporal heterogeneous under-reporting bias. There is evidence of an average of 4.5 days from farmers noticing H5N1 outbreaks and reporting, and the infected premises were subsequent depopulated [14].

Fasina et al. [15] completed a full analysis of the risk factors associated with highly pathogenic H5N1 influenza in the poultry farms in Nigeria during the 2006–2007 epidemic. They found the top three risk factors for transmission of avian influenza were, (1) receiving visitors on farm premises, (2) purchasing of poultry, and (3) farm workers that lived off the premises. The factors identified to be important for the dissemination of the highly pathogenic, H5N1 influenza in poultry include: multiple species in backyard poultry [16], the number of backyards with poultry [17], poor biosecurity and proximity to wild birds [18], and proximity to infected farms [19]. In addition, proximity to the highway network appears to promote epidemic dispersal in Nigeria [20]. Ekong et al. [21] show that the immediate challenge for the H5N1 outbreak in Nigeria was the control of outbreaks in backyard and small-scale poultry flocks.

Public health works, veterinary officials, and policy makers could benefit from a standard methodology that can quantify the risk factors in future emerging epidemic and help guide the design of surveillance networks. We will present a framework for constructing an optimal spatial surveillance network that is usable by policy makers based on multi-scale, multi-host epidemic models with rigorous experimental design, sensitivity analysis, uncertainty quantification, risk analysis and optimization over a chosen metric. An optimal surveillance network could reduce cost of surveillance needed, while providing much better coverage.

A rigorous biosurveillance an for zoonotic outbreaks must consider multiple host species, including animals and humans. For the mathematical model to be useful in guiding the plan, it must be multi-scale, considering both local and long-distance effects and allowing for multiple points of entry for a pathogen. In the absence of specific data on the transportation of poultry through trade in Nigeria, we assumed a spatial spread between counties that falls of exponentially with the distance between counties, with the expectation that this is likely to capture important features. The importance of spatial and temporal heterogeneity of livestock transport in disease surveillance has been noted [22]. Since with more complicated multi-host, multi-scale models there is uncertainty in mechanisms and parameters, it is important to include stochasticity in the model, both in movement of the infection and in model input parameters.

All models will have some uncertainty in the parameter values, initial conditions, and the effect of the factors not included in the model. That is, the model must consider a range of forecasts as the parameters vary through a possible range of values for the pathogen or suite of pathogens chosen. In our simulations, we used an experimental design, with Latin hypercube sampling, to quantify the forecasts over a suite of possible parameter values. The uncertainty analysis captured both the intrinsic (due to stochasticity) and extrinsic (due to parameter values) uncertainty in the model output. Sampling the parameters over their entire range of possible combinations quantified the uncertainty of the mean behavior of the system, and to explore the whole possible range of forecasts. We then applied sensitivity to rank the relative importance of the input parameters on the model forecasts. To complete the analysis, we used spatial statistics to rank the network nodes (counties, LGAs, etc.) based on the risk of infection given an outbreak.

Using the uncertainty analysis as a guide, we demonstrate two methods for constructing a biosurveillance network based on the risk map, using optimization to determine the 10 nodes needed to either maximize probability of detection or maximize the speed of detection. We then validate the county risk map with data from a known outbreak, confirming that the outbreak would likely be detected based on the risk map. Although this framework and methodology was used for optimizing the biosurveillance network in our specific model, the approach is general and can be applied to other geographical regions, species, and pathogens, and maximizing the effectiveness from a combination of cost and detection perspectives.

In the following sections, we will develop a multi-scale and multi-host epidemic model with a statistical treatment for uncertainty of parameters as a planning tool for active surveillance for a zoonotic disease. To evaluate the potential of epidemiological modeling in planning of a surveillance network in poultry, we simulate outbreaks of highly pathogenic avian influenza H5N1in poultry populations in Nigeria. These simulations illustrate how epidemiological modeling can assist in planning for biosurveillance prior to a disease arriving in a country (monitoring based on risk analysis).

## Materials and Methods

### Epidemic model

We begin with a stochastic two-stage hybrid model of the spread of a multi-host infectious disease applied to highly-pathogenic H5N1 influenza among agricultural poultry, backyard ducks, wild birds, and humans in Nigeria. Nigerian local government areas (LGAs) were used as the fundamental nodes in the disease transmission network. The two stages of the model encompass stochastic disease transmission between LGAs as well as small-scale dynamics of disease spread within a LGA. Disease transmission between LGAs was based on the number of infected animals in the infectious LGA, the number of susceptible animals in the uninfected LGA and the distance between the locations. The internal (intra-LGA) dynamics of disease spread were modeled using a distribution of solutions to deterministic differential equations with parameters sampled from ranges of values selected for uncertain disease and response parameters. For additional specifics of our epidemic model see [23]. The model is designed to be as general as possible so that it can be adapted to varying parameter values and situations [23]. Our modeling framework and approach can also be applied to other epidemic and spatial models provided they are capturing the important aspects of disease transmission, progression, and spatial spread.

### Inter-Patch Disease Transmission Model

We have developed a general two-stage mathematical model that describes the spread of an infectious disease in a multi-species susceptible host population. The top-level (“inter-patch”) model is a stochastic simulation where each node is a “patch” which is a geographic or epidemiological unit where the uniform mixing approximation (i.e. homogenous inter-host contact rates) holds. At time *t* during the simulation, if a particular patch *X* is susceptible (i.e., free of infected hosts), its probability of becoming infected at time *t* is given by

$$p_{X}\left(t\right)=1-\exp\;\left[-\Gamma_{X}\left(t\right)\right]$$ (1)

The kernel function describing propensity for infection is given

$$\Gamma_{X}(t)=\sum_{Y=1}^{N_{I}(t)}\sum_{\alpha }\eta_{\alpha }(X,Y,t)[\chi_{S}^{XY}(t)\kappa_{S}(d_{XY})+\sum_{m}\chi_{m\alpha }^{L}(t)\kappa_{m\alpha }^{L}(X,Y)]$$ (2)

where *Y* is the index over all infected (i.e., with latent or symptomatic infected hosts) patches at time *t*, *N*_*I*_ *(t)* is the number of patches with infected hosts at time *t*, and *α* labels the host species type. The function *η*_*α*_(*X*, *Y*, *t*) in Eq (2) is the time-dependent risk of infection given full (i.e., optimal) contact of patches *X* and *Y*,

$$\eta_{\alpha }(X,Y,t)=\sum_{\alpha^{'}}[\beta_{\alpha \alpha^{'}}^{AS}(X,Y)A_{\alpha^{'}}^{Y}+\beta_{\alpha \alpha^{'}}^{IS}(X,Y)I_{\alpha^{'}}^{Y}+\beta_{\alpha \alpha^{'}}^{LS}(X,Y)L_{\alpha^{'}}^{Y}+\beta_{\alpha \alpha^{'}}^{BS}(X,Y)B_{\alpha^{'}}^{Y}+\beta_{\alpha \alpha^{'}}^{ES}(X,Y)E_{\alpha^{'}}^{Y}]\;S_{\alpha }^{X}$$ (3)

and the five terms in Eq (3) give the transmission rate between infective hosts in different disease states in *Y* to susceptible hosts in *X*. The coupling strengths are given by the parameters $\beta_{\alpha \alpha^{\prime }}^{MN}\left(X,Y\right)$ where *M* and *N* are disease states and transmission is from species α’ in disease state *N* to species α in disease state *M*. The time evolution of the disease states are outputs of the lower level (“intra-patch”) model, which, within a single patch, models disease transmission between individual hosts and disease progression. The intra-patch model will be discussed in the next section.

The dependence of infection risk on the host contact/transport network of the inter-patch model is introduced through the factor in brackets in Eq (2). The first term gives the distance dependence of contact due to local, “area spread”-type transmission modes. The short-distance movement kernel is given by

$$\kappa_{S}(d_{XY})=exp[-d_{XY}/r_{S}]$$ (4)

where *d*_*XY*_ gives the surface distance between the geographic centroids of *X* and *Y*. The length scale *r*_*S*_ is the disease-specific length scale of transmission, to be obtained from data or the literature. Transmission occurs via direct contact between regions *X* and *Y*.

The second term in brackets of Eq (2) is the long-distance (e.g. animal shipments, human travel, etc.) transport term and is expressed as

$$\kappa_{m\alpha }^{L}(X,Y)=1-exp[-bN_{m\alpha }^{L}(Y\to X,t-\Delta t,t)]$$ (5)

The index *m* labels the particular transport mode being considered. For instance, it is possible to differentiate between transport due to airplanes, trains or trucks. The function $N_{m\alpha }^{L}\left(Y\to X,t-\Delta t,t\right)$ gives the number of hosts of species *α* transported from *Y* into *X* by transport mode *m* over a time interval Δ*t*.

### Intra-Patch Transmission/Progression Model

The function *η*_*α*_(*X*, *Y*, *t*) in the inter-patch model gives the infection risk of patch *X* from patch *Y* given optimal contact. To produce this risk, we must know at any time *t* in the simulation the number of infected hosts in *Y*, their degree of infectivity, the number of susceptible hosts in *X*, and their degree of susceptibility. We therefore require a model of intra-patch disease transmission and progression that can be used for calculating the number of infected hosts in an infected patch *Y* at time *t*.

In the intra-patch model we simulate disease transmission/progression using a SIR-like mathematical model of coupled nonlinear ordinary differential equations. The model features discrete compartments that represent the particular disease state, coupled by exponentially distributed transition rates. If patch *X* is deemed infected in the inter-patch simulation, an initial number of susceptible hosts in the multi-species host population are converted to asymptomatic, infected hosts. This initial seed is typically not smaller than ~100 individuals order to keep out of a temporal regime at the start of an epidemic where the uniform mixing approximation breaks down. The intra-county model then describes the dynamics of transmission and progression within a location X, such as a county or other government-defined area, incorporating a response and mitigation architecture.

### Data sources

**Fig 1:**
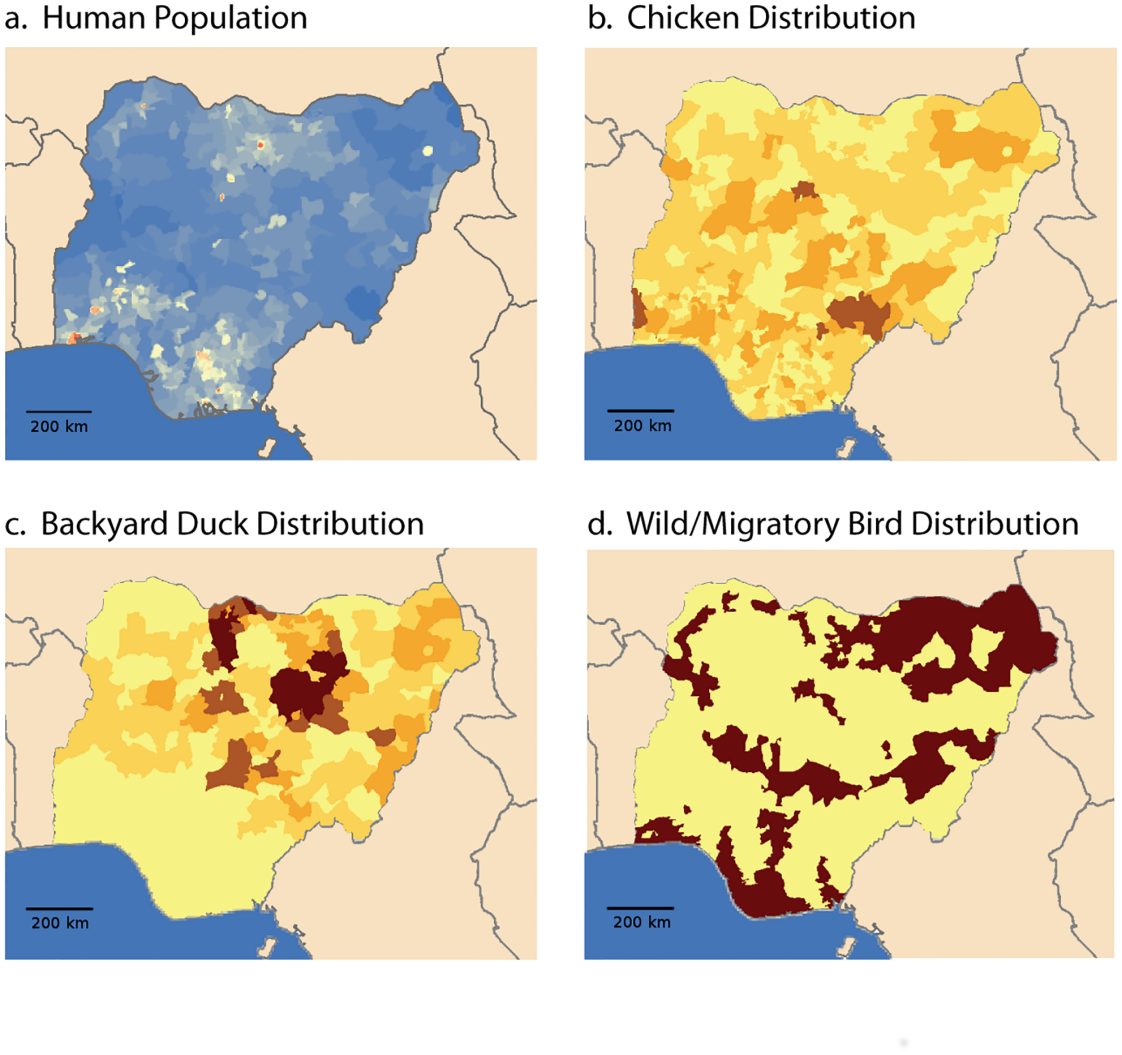
Shown are the distribution of the population of people (a), Chickens (b), backyard ducks (c) and migratory birds (d) used in these simulations. Colors are relative and intended to illustrate the population distribution for specific species, with darker indicating greater numbers. The human population is shown progressing from blue to red, with red indicating greater numbers. Data was gathered largely from United Nations sources and was processed to allow association with specific Nigerian local government associations (LGAs).

**Fig 2:**
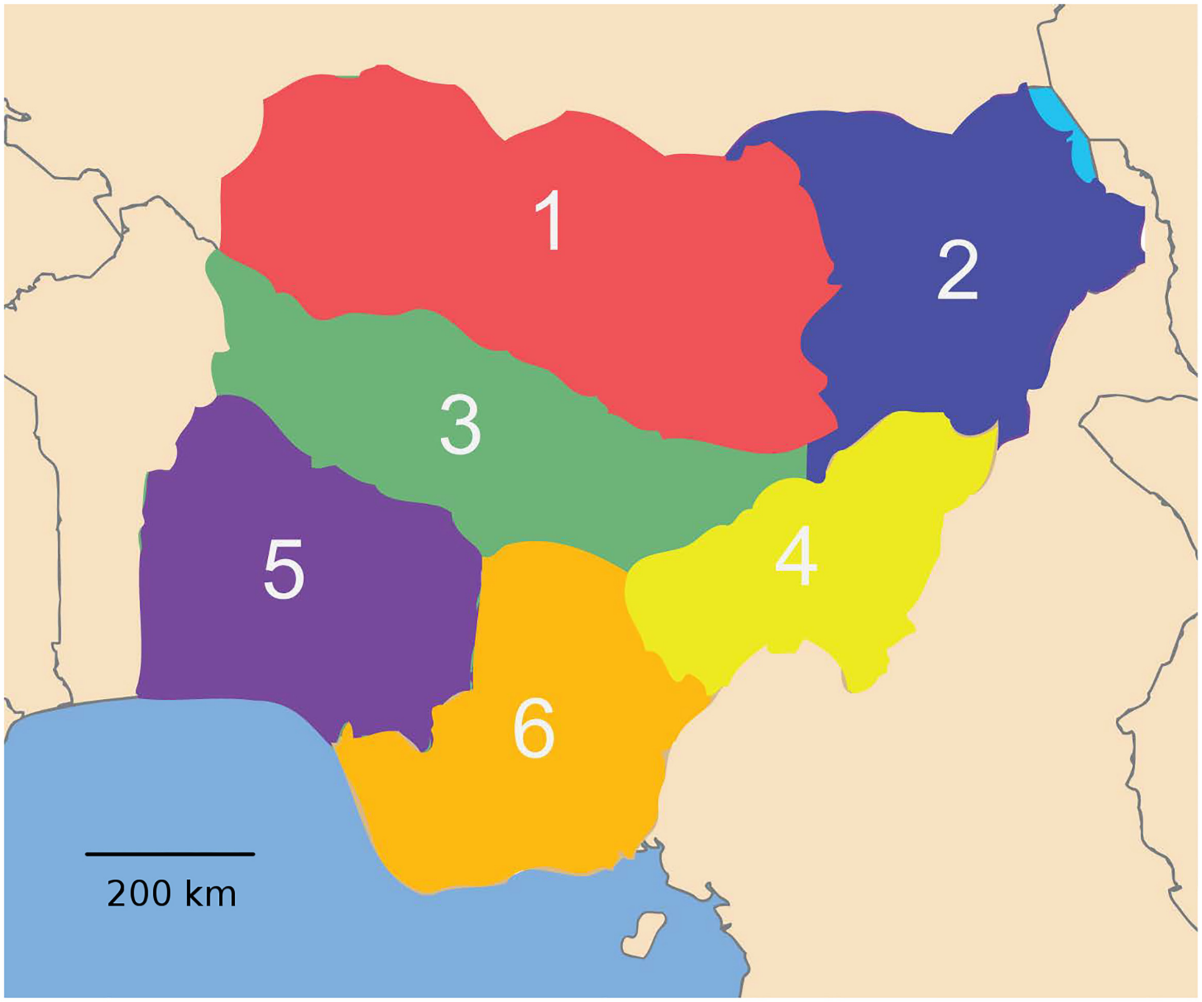
Nigeria was divided into six regions that were used in the experiment design to test for geographic differences for the start of epidemics.

Information on the geographical distribution of animal and human populations was obtained from a number of publically available sources, with an emphasis on rapidly accessible and freely transferable information. We focused our initial efforts on data as close as possible in time to the actual outbreak, while also desiring data that is frequently updated and with worldwide extent. The four main populations used in this study included humans, chickens, backyard ducks, and wild or migratory birds (Fig 1). Backyard ducks, in this case, imply duck populations in small-scale or household poultry production systems and were included due to their importance in Nigeria, particularly in the northern half of the country. The majority of this data was available through the Food and Agriculture Organization of the United Nations (UN-FAO), including raster graphics of chicken population “Gridded Livestock of the World 2007” [24], raster graphics of human population “Gridded Population of the World, version 3) (CIESIN, 2005), and the global lakes and wetlands database [25], from which a rough estimate of potential migratory bird locations was derived. The backyard duck populations were derived from data from the Nigerian National Bureau of Statistics [26,27]. Nigeria was divided into six regions that were used in the experiment design to test for geographic differences for the start of epidemics (Fig 2).

### Experimental Design and Analysis

The experimental design analysis is based on a representative sample of possible epidemics and consequences for evaluating the performance of surveillance plans. Each ensemble of model simulations, for a fixed set of parameter values, captures the intrinsic stochasticity in the forecast. Multiple simulations of the epidemiological model at different sets of parameter values provide both variation due to sampled parameter values and modeled inherent stochasticity. We use a Latin hypercube sample (LHS), with an underlying orthogonal array of strength three [33,34,35], to uniformly sample the feasible parameter space. This approach addresses variability due to uncertain parameters for stochastic models and allows identification of main effects of individual inputs without bias from two factor interaction effects. Sensitivity analysis of the response is used to rank the importance of the input parameters [28,29] and quantify their variation created by the model stochasticity.

Seven input parameters and reasonable parameter ranges were specified (Table 1) to generate variability in disease forecast and mitigation parameters. The location of the initial outbreak is analyzed as an eighth parameter. The disease input parameters sampled in the experiment are subclinical and clinical stage times. These were chosen due to inherent biological uncertainty and based on previous studies showing result sensitivity with these parameters [30]. For example, the average time of both subclinical and clinical disease stage period in days varies greatly with what is known about H5N1 influenza in birds and human. The average duration that animals are in each stage may also change as the epidemic proceeds and antigenic drift creates different virulence and disease characteristics [31]. The remaining inputs sampled for the experiment dealt with possible scenarios for response to an epidemic: quarantine policy, short-range movement control, time after infection and number of infected birds that triggered culling. A sample of 40 candidate locations for outbreak start was chosen in order to ensure good coverage, and representation of both geographic and species population characteristics, in the population of 220 LGAs. The 40 outbreak start locations were selected to maximize the minimum pairwise distance between points [32], a space-filling experiment design strategy commonly used for selecting representative inputs to propagate in a deterministic model and useful for stochastic models as well.

**Table 1:** Biological and control measure model parameters used for highly pathogenic H5N1 in Nigeria.

Model parameters	Poultry
Transmission Rate/subclinical Animal $\left(t_{\alpha }^{A}\right)$	0
Transmission Rate/Clinical Animal $\left(t_{\alpha }^{I}\right)$	0.000000425
Transmission Rate/Seroconverted non-progressing animal $\left(t_{\alpha }^{L}\right)$	N/A
Susceptibility per animal (*S*_*α*_)	1.0
Subclinical stage residence time (days) (*1/λ*_*L*_)	1–3
Clinical signs stage residence time (days) (*1/λ*_*I*_)	1–1.5
Seroconverted stage residence time (days) (*1/λ*_*C*_)	N/A
Infected animals that progress to clinical signs (fraction) (*1-θ*_*L*_)	1.0
Infected animals that dies (fraction) (*θ*_*D*_)	0.975
Recovery stage residence time (duration of immunity)	Indefinite
Vaccine protection efficacy for susceptibles $\left(\varepsilon_{V_{S}}\right)$	N/A
Vaccine protection efficacy for infected animals $\left(\varepsilon_{V_{e}}\right)$	N/A
Culling rate (animals per day) (*ε*_*C*_)	53,500
Quarantine policy (efficacy)(*ε*_*q*_)	0–1.0
Vaccination policy	N/A
Inter-state movement control efficacy (fraction) (*χ*_*L*_)	N/A
Short-range movement control efficacy (fraction) (*χ*_*S*_)	0–0.5
Radius of surveillance zone (miles)	6.2
Time between decision and quarantine (county level-days) $\left(\tau_{Q}^{X}\left(begin\right)-\;\tau_{D}\right)$	2.67 if not in surveillance zone.1 if in surveillance zone.
Time between detection and culling (county level-days)$\left(\tau_{C}^{X}\left(begin\right)-\;\tau_{D}\right)$	5.67 if not in surveillance zone.1 if in surveillance zone.
Time between detection and vaccination (county level-days) $\left(\tau_{V}^{X}\left(begin\right)-\;\tau_{D}\right)$	N/A
Characteristic length of local speed (miles) (*r*_*0*,_ *r*_*S*_)	5.53

The uncertainty analysis within the experiment design approach provides statistical estimates for the correlation between the spatial locations. The variation in the simulated epidemics is due to inherent stochasticity modeled in the intra-patch and inter-patch spread, the variation in the sampled parameter values, and initial infected locations in the experiment design. The responses for sensitivity study include assessment of the disease progression (day infection appeared in a location, duration of the epidemic) and the induced extent of consequences (for example total infected birds). The experimental design provided a basis for understanding the relationship between responses of interest and disease characteristics or potential mitigations that drive an outbreak and to characterize uncertainty in the outbreak response due to the various sources of possible variation, including key input factors, such as disease characteristics or intrinsic sources of variation, such as movement between LGAs.

A designed experimental plan specifies a sufficient number of simulation runs to enable an effective basis to achieve analysis goals [33]. The study sampled 40 starting locations for the epidemic that are representative of geographic and species population characteristics. Each starting location was coupled with 32 variations of the seven disease and control factors to evaluate their impact. The seven factors were sampled according to an orthogonal array (OA) based Latin hypercube sample (LHS), with an underlying orthogonal array that was a fractional 2-level factorial design of strength three for 7 factors. This type of experiment allows identification of main effects of individual inputs without bias from two factor interaction effects and is a good basis for identifying a few primary important factors [28,29]. The resulting simulation study included 1280 (40 x 32) simulation runs and was adequate to identify effects of control factors including differences in response effects due to start location. The LHS experiment plan also achieves a representative range of inputs that, along with model stochasticity, results in a viable basis for evaluating the distribution of responses, i.e., uncertainty analysis [34–37]. The 1280 simulated epidemics form a basis for evaluating the distribution of a response of interest that includes not only propagated variation in parameter settings but run-to-run (intrinsic) variation as well. Likewise, the evaluation of influential parameters based on analysis of this data will be impacted by run-to-run variation in addition to variation due to parameters.

The sensitivity of the response, or model outcome, to an input factor was evaluated by a standard goodness of fit measure, namely *R* ^*2*^, based on fitting a single input, *x*, to a linear response modeled as *Y = mx+b*. This is not to say that the relationship between the responses of interest and the parameters varied is linear but this is a first-order evaluation of sensitivity related to a variance based sensitivity analysis for a first order model.

An alternative approach also evaluated R^2^ for an analysis of variance for a single parameter as treatment with high or low values. Both these first order models were deemed appropriate for the limited number of runs used to vary 7 parameters and due to the fact that the underlying orthogonal array had 2-level values for each parameter. The relative importance of the input parameters is assigned based on the values of *R* ^*2*^ over the estimated parameter input ranges. An input *x*_*1*_ is judged a better predictor (more influential) than x_2_ for a given response if *R* ^*2*^ *(x*_*1*_ *)>R* ^*2*^ *(x*_*2*_ *)*.

The input parameters, such as time to cull or quarantine efficacy, that most impact the course of the epidemic can be used to help guide promising effectiveness of control, even in the presence of uncontrolled factors related to disease stages and intrinsic variability. That is, this approach will quantify the importance of the input parameters in mitigating the epidemic. The increment in *R* ^*2*^ for fitting an additional factor is be used as the basis for evaluating relative importance of inputs after accounting for a common factor, such as start location. The conclusions for the two approaches (linear regression or analysis of variance based on high or low parameter values) to sensitivity analysis were consistent and used to provide insight to effective graphical presentation of results.

The experiment design efficiently generates a broad distribution of potential epidemic scenarios to evaluate and compare strategies for surveillance planning. These samples provide the basis to evaluate mitigation strategies with respect to the parameters. The sensitivity analysis accounts for the variability in the forecasts resulting from both the parameter variation and model stochasticity. The experimental design analysis also serves as the basis for factorial and fractional factorial design without replication to quantify the sensitivity of the input parameters [36].

Our analysis is sensitivity analysis is first-order. Higher order analysis for estimating specific elements of a more complex statistical model, including second-order or higher parameter effects and individual components of variation, would require significantly more simulation runs.

To evaluate the simulation results with respect to the 2006–2007 H5N1 epidemic, we calculated the Getis-Ord Gi* statistic (“hot spot analysis”) for the number of times out of 1280 model simulations an LGA was positive for H5N1 [38]. This allowed us to identify LGAs that were either more positive (“hot”), more negative (“cold”) or similar to neighboring LGAs. We determined the neighborhood size by measuring the distance at which spatial autocorrelation was maximal The Moran’s I derived Z score was highest (I = 12.74) at 200 km. We therefore used a fixed distance band of 200 km to assess the Gi* statistic for each LGA. Which is to say, LGAs beyond 200 km from a given LGA were not included in a given hot spot analysis iteration. To quantitatively assess the nature of our modeling results compared to the 2006–2007 epidemic data, Gi* Z scores were regressed (generalized linear model) against the number of times each LGA contained an H5N1 epidemic (a Poisson variable).

Finally, we used the following methodology to design a surveillance architecture for the early and effective detection of outbreaks that balances two criteria: speed of detection and probability of detection. First, a collection of simulated epidemics was generated using the design criteria described above. In this case, 1280 simulations were run covering both disease parameter and release location space. Second, a collection of appropriate locations for surveillance was gathered. In this case, the local government area (LGA) with the largest animal population within each Nigerian state was considered. Since the autocorrelation distance determined above of 200 km is larger than the typical size of the 35 Nigerian states, this assumption greatly reduces the size of search-space of possible surveillance architectures, while making it quite likely that a near-optimal solution will be found. Third, the time for the disease to reach each proposed surveillance location was calculated for each simulated epidemic. If the time required was longer than 20 days (when optimized for probability of detection) or longer than 5 days (when optimized for speed of detection), the time required was simply set to 30 days as a penalty for non-detection. These time limits were determined through trial and error, and may be adjusted to fit other problems of interest. Finally, the average time for the disease to reach a proposed surveillance location was calculated for all possible surveillance architectures, including penalties. The surveillance network that minimized the average detection time could then be calculated for both probability of detection, and the speed of detection in Nigeria. This approach was possible due to the reasonably small scale of this problem, but could be adapted to more sophisticated optimization algorithms when appropriate.

## Results

### Sensitivity Analysis

Sensitivity assessed on the basis of R^2^ values for fit of single covariates, including the seven disease and control factors as well as factors associated with start location, show that generally, for the responses of interest, start location leads to important variation in the response, followed by time to cull and trigger number. The increase in R^2^ values with fitting seven disease and control factors in addition to effects due to start location, indicated as well that time to cull and trigger number are important. These observations suggest that the days from the detection trigger to the time to start culling and the number of animals required to trigger the onset of culling are the most important parameters to consider for affecting disease control.

**Fig 3:**
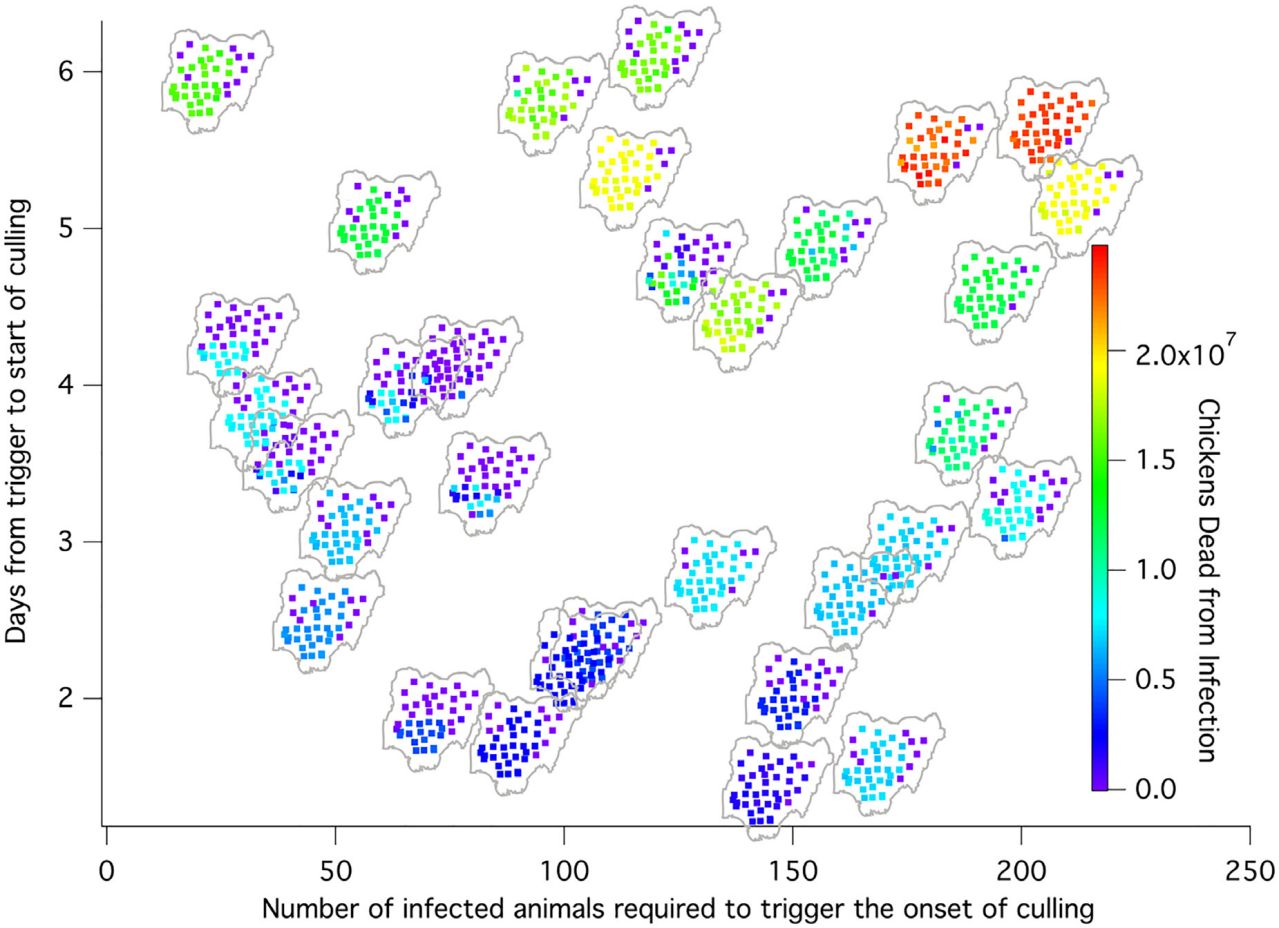
The impact on the total number of chickens that died from infection as the two most significant simulation parameters are varied, the time before initiation of culling vs. the number of infected animals required to trigger the onset of culling. The maps are color-coded for the number of chickens dead from infection.

**Fig 4:**
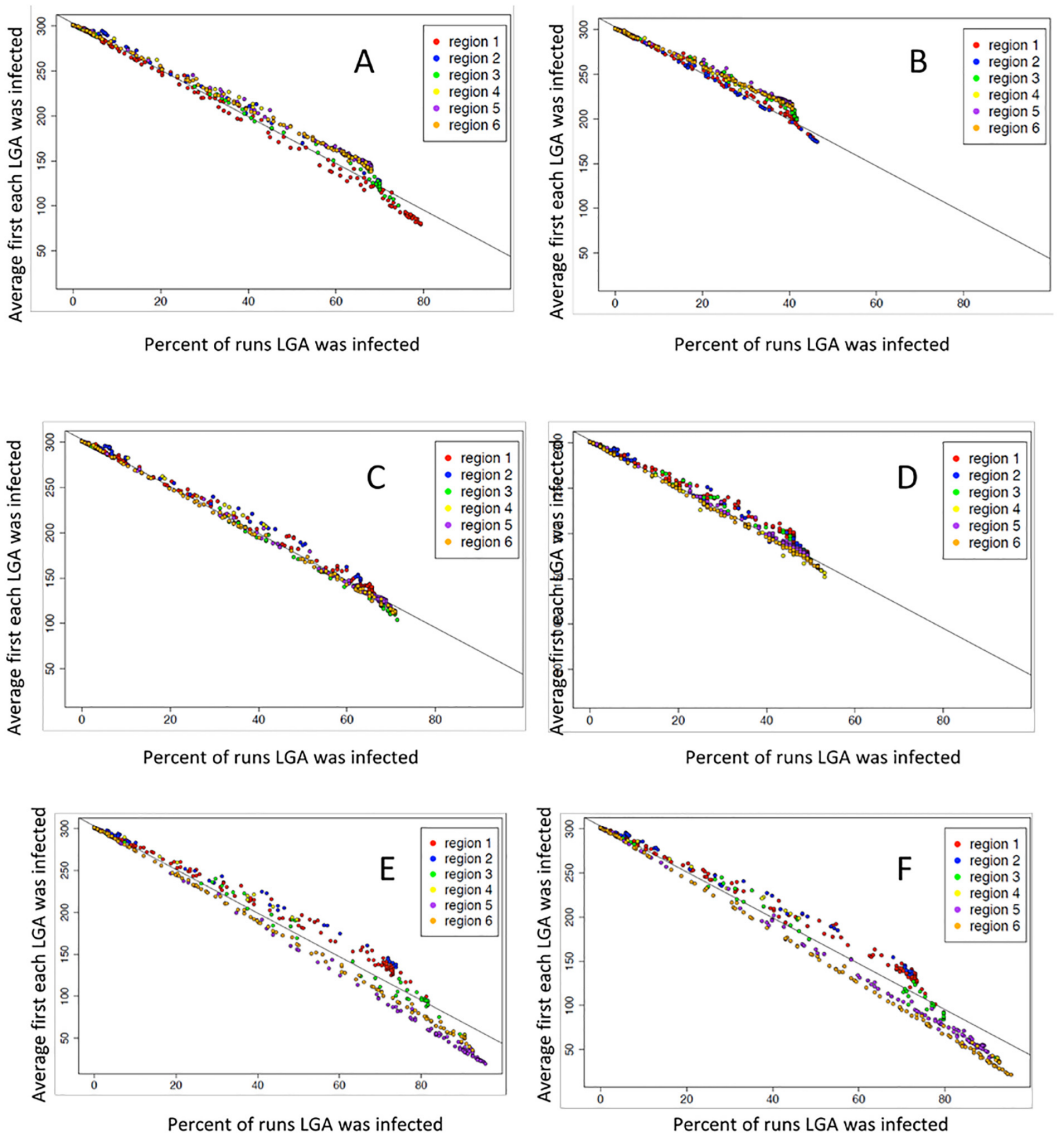
Local Government Associations (LGA) over all simulation runs for the average number of times that each LGA was infected for all the simulations and the average first day a LGA becomes infected for each region where the epidemic is started. Epidemic starts in region A) 1. B) 2, C) 3, D) 4, E) 5, F) 6.

Over the 1280 runs, the analysis showed a relationship between the proportion of runs that a Local Government Association (LGA) is infected and the first day of infection of a LGA, for runs initiated in one of the six candidate regions (Fig 3). For some responses, short-range movement control and time to quarantine are important. The results of the 1280 runs show the importance of geography of where the epidemic starts (Fig 4). Epidemics that are started in regions two and four have less overall impact as far as LGA infected but also have much shorter epidemics (Fig 4).

### Simulation Results

A total of 1280 simulation runs were performed that included 32 sets of parameters for each of 40 release locations. The total number of chickens that died from infection was monitored as the key model outcome to give a measure of epidemic size as a function of the simulation parameters. Fig 3 shows results from all the simulations on the impact on the total number of chickens that died from infection as the two most significant simulation parameters are varied, the time before initiation of culling vs. the number of infected animals required to trigger the onset of culling. The time before initiation of culling and the number of infected animals required to initiate the onset of culling were determined to be the most important parameters through sensitivity analysis. Spatial information is presented in Fig 3 by creating a slight offset, linear in the latitude and longitude for each release location. As such, the impact of both release location and the important mitigation parameters can be seen.

We find that the size of the eventual epidemic is a strong function of the

1. number of infected animals required to trigger the decision to begin culling (smaller is better),
2. delay before the commencement of culling operations (shorter is better), and
3. location of the initial outbreak.

Fig 3 illustrates the impact of release location on the eventual epidemic size for all possible outcomes based on the uncertainty of both disease characteristics and potential mitigations. Each of the 1280 simulation runs performed are shown, including 32 sets of parameters for each of 40 release locations. Individual maps were created demonstrating the impact of release location on the eventual epidemic size for each of the 32 simulation parameter sets. These maps, organized by parameter set in Fig 3, allow spatially resolved details of the simulations to be determined. In particular, the highest impacts are seen from outbreaks initiated in the southwest of the country, where the populations of all of the species is the highest. The impact of release location is more important when surveillance and response efforts are effective at trapping the disease (parameter sets in the lower left of Fig 3) than when they are ineffective at stopping the disease from spreading throughout the country (parameter sets in the upper right quadrant of Fig 3).

### 2006–2007 Epidemic Comparison

**Fig 5:**
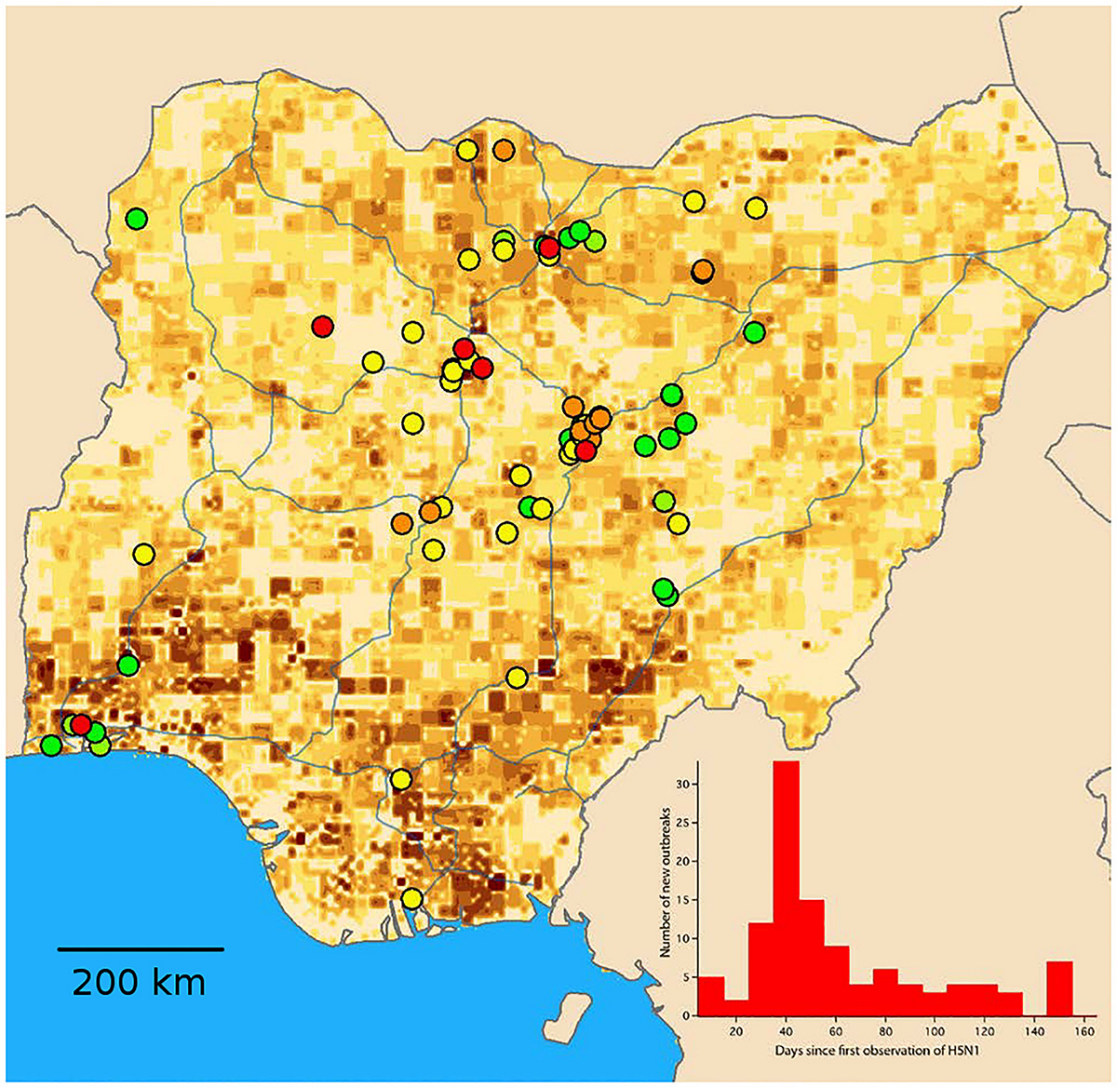
Fig 5 shows a depiction of the 2006 Nigerian avian H5N1 influenza outbreak in 2006. The data shown are structurally similar to that the data acquired from the EMPRES-i system developed by the UN-FAO. Inset is a histogram of the outbreak frequency, showing a peak at approximately 40 days post the initial identification of the disease in Kaduna, Nigeria.

Fig 5 of the 2006 Nigerian avian influenza outbreak is structurally similar to the data acquired from the EMPRES-i system developed by the UN-FAO. The disease was first identified in a commercial farm in Kaduna state, in north central Nigeria, on the 10th of January 2006. The spread of the epidemic is color-coded, with red representing outbreaks observed within two weeks of the index case, orange from 2–4 weeks, yellow from 4–6 weeks and green past this timeframe. The disease was concentrated in the chicken and backyard duck populations in north-central Nigeria, though outbreaks were observed in the heavily populated city of Lagos, in southwest Nigeria. It was initially thought that long-range transmission was responsible for the appearance of the disease in Lagos. The appearance of the Lagos cases caused the Nigerian authorities to doubt the effectiveness of the north-south transportation surveillance and quarantine efforts that were put in place soon after the beginning of the outbreak. It was later shown through phylogenic analysis that the new cases were the result of an independent introduction of the disease [39].

**Fig 6:**
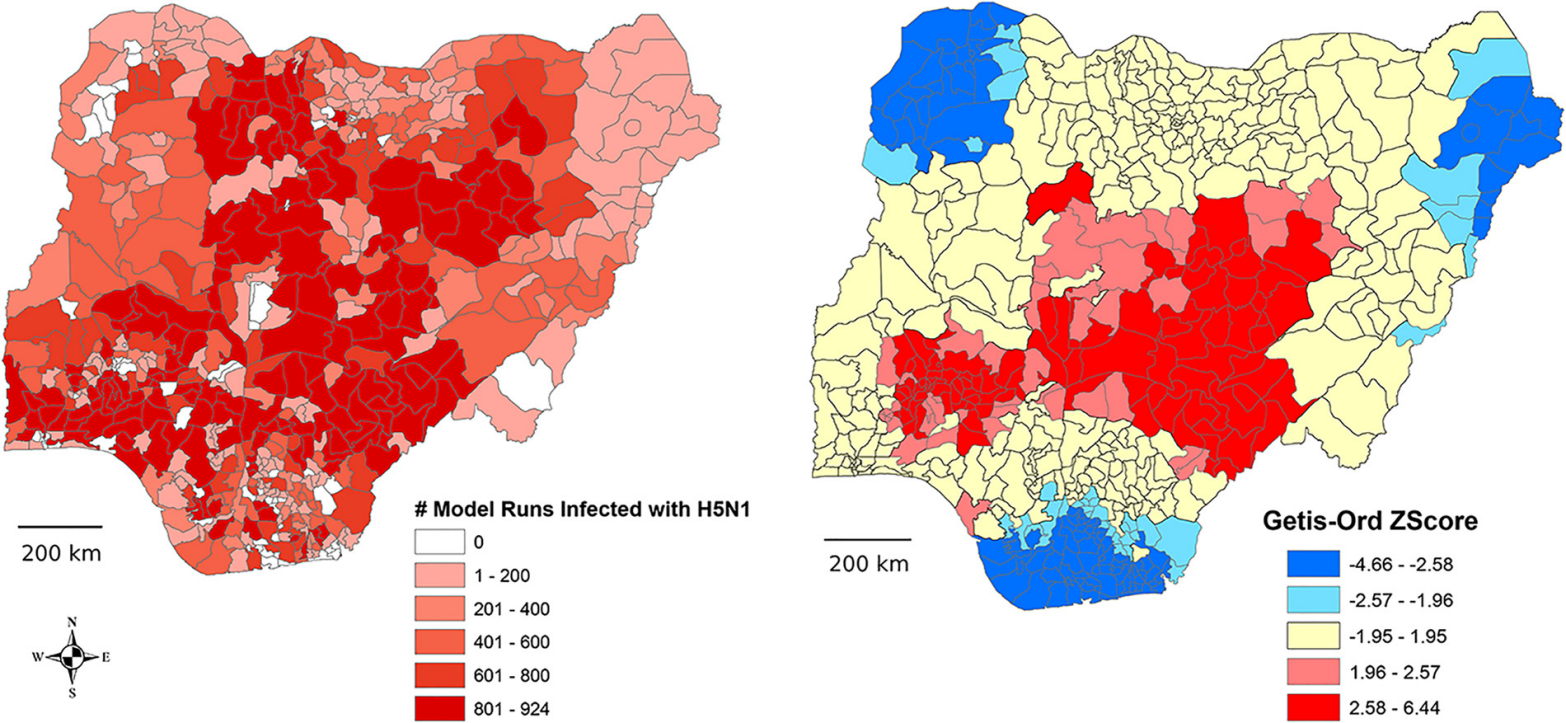
Validation of modeling results. A) The number of times a Local Government Association (LGA) was affected out of 1280 model simulations. B) Map overlay of 2006 H5N1 epidemic locations (black dots) with Z scores resulting from the Getis-Ord Gi* statistic as applied to model output. LGAs colored red are statistically “hotter” (more positive model simulations) while LGAs colored in blue are statistically “colder” (fewer positive model simulations) than their neighbors within a 200 km moving window. The relationship between simulated data and 2006 epidemic data was strong in that the number of times a LGA was affected by H5N1 in 2006 directly related to Gi* Z score values (χ2 = 74.82, df = 1, P < 0.0001).

Fig 6 shows each LGA plotted and scaled by the number of times it was infected over the 1280 runs. The middle of the country contains a high presence and probability (Fig 6) of infection, which follows the H5N1 epidemic of 2005–2006 [15,20]. The hot-spot analysis, based on the current simulation data, (Getis-Ord Gi* statistic) highlighting the LGAs with the highest (Z > 1.96) and lowest (Z < -1.96) risk for becoming infected during influenza outbreaks. We then show how this was spatially related to real epidemic data (Fig 6). The relationship between simulated data and 2006 epidemic data was strong in that the number of times a LGA was affected by H5N1 in 2006 directly related to Gi* Z score values (χ^2^ = 74.82, df = 1, *P* < 0.0001). That is, as H5N1 infection events in a LGA increased in 2006, so too does probability that the LGA was a simulated hot spot, as indicated by Z value; more H5N1 events tended to occur in simulated “hot spots”. This finding indicates that simulated risk (as indicated by Z score) and real data statistically significantly spatially overlap.

Getis-Ord Gi* results can be observed in Fig 6. There were three cold zones containing LGAs with Z scores < -1.96 and two hot zones comprised of LGAs with Z scores > 1.96. Cold zones included 100 and 36 LGAs that were one or two+ standard deviations, respectively, lower than the mean number of positive model runs. There were 45 and 97 LGAs in the hot zones that were one or two+ standard deviations, respectively, above the mean number of positive runs for the entire study site (Nigeria). The LGA that was most different from its neighbors in terms of the number of times it was positive for H5N1 was Makurdi (central Nigeria), with a Z score of 6.44. The “coldest” LGA was Degema in south-central Nigeria with a Z score of -4.66.

**Fig 7:**
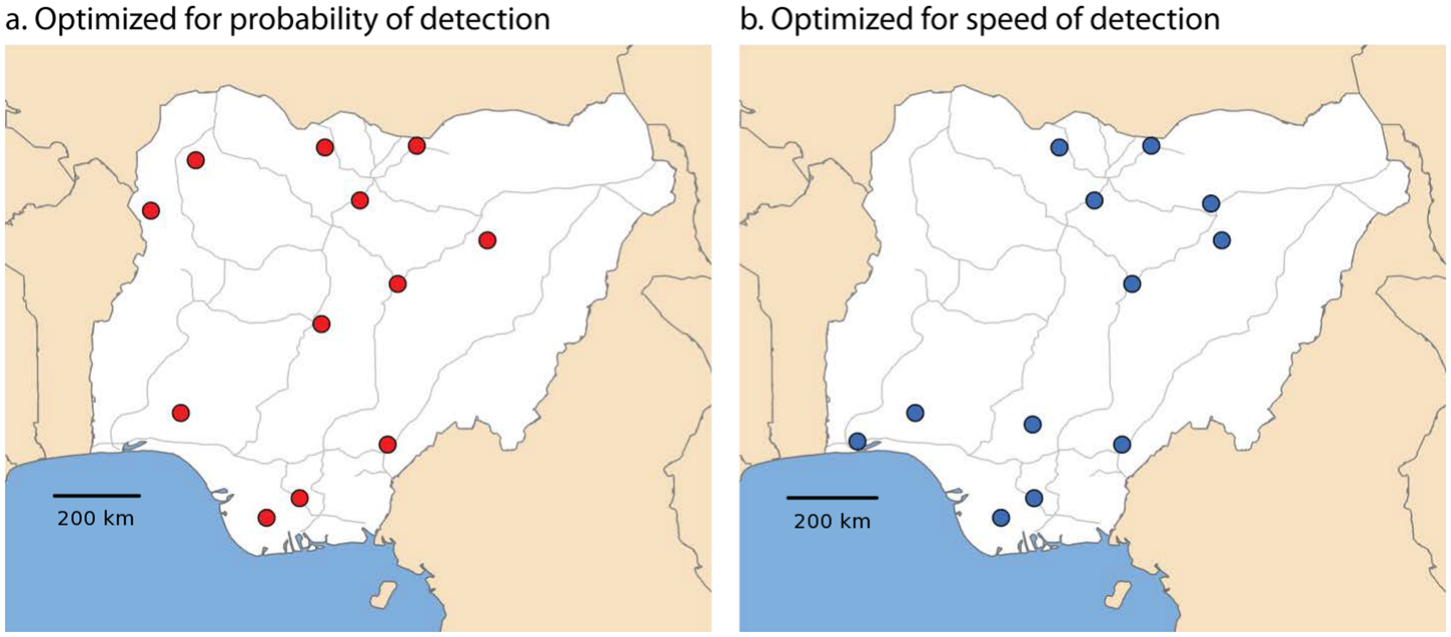
Surveillance architecture optimized for A) probability of detection, and B) speed of detection in Nigeria. When optimized for probability of detection, surveillance stations are dispersed throughout the country. When optimized for speed of detection, two groups surveillance stations form, one centered on the large population of animals in the north and one centered on the large population of animals in the south.

This study optimized the surveillance network based on a specific balance between optimizing the speed and probability of detection of an emerging epidemic. The approach is flexible and could be used for other weightings between these criteria. The methodology employed in the surveillance architecture was simple, but easily extensible to specific problems of interest (Fig 7). Although the surveillance architecture shown in Fig 7 was designed for a specific outbreak, it can be easily generalized for other similar emerging epidemics.

## Discussion

Surveillance will become increasingly important in detecting and reducing the impacts of the disease as avian influenzas continue to emerge and evolve from the interplay between wild birds, poultry, and humans. However, as Rich et al. [40] eloquently point out, in animal disease outbreaks, where human sociology can actively influence the epidemiology of disease, it is critical to evaluate resource allocation simultaneously. That is, we must evaluate how human behaviors influence the epidemiology of disease, and consequently, the cost-effectiveness of surveillance systems over time. We build on the framework for animal disease monitoring given by Rich et al. [40] for allocation and composition of surveillance coupled with disease biology, to include all possible scenarios and geography.

In addition to wide range of risk factors for the spread of diseases in animals, geography has been shown to be a primary determinant of the size and timing for epidemics [41]. For example, many disease outbreaks will be dependent on the geography of a regions through vectors or other geographically determinants [42–44]. For H5N1, proximity to the highway network and epidemic nodes appeared to promote epidemic dispersal in Nigeria [20,45].

In the 2009 H1N1 influenza pandemic, the pathogen emerged and changed quickly as it progressed and moved globally [46,47]. The reasons behind the rapidly changing H1N1 disease characteristics, reporting bias, and medical response differences may never be fully understood. Therefore, planning for future biosurveillance will need to incorporate all possible scenarios for an epidemic based on the range of potential disease characteristics, coupled with a method for determining the most likely locations for the disease to place surveillance activities with limited resources. For planning of biosurveillance structure, a systems dynamics approach of human-animal interface, range of potential diseases characteristic of an emerged disease, coupled with the spatial geography would provide the most informative framework for decision makers and resource allocation.

As highlighted by Childs and Gordon [48], surveillance and mitigation planning can, and should begin prior to an infectious disease spreading to a region, thus greatly increasing the probability of detecting the virus and reducing its impact. In another analysis, Cannon [49] reviewed several metrics of surveillance resource optimization problems based on the various objectives by decision-makers such as maximizing detection, minimizing detection time, and maximizing the benefits from early detection. Determining optimal surveillance networks for an emerging pathogen in humans or animals is difficult since it is not known beforehand what the characteristics of a pathogen will be or where it will emerge. Our framework for surveillance planning incorporates the range of disease characteristics possible using a rigorous experimental design, and geography to best optimize limited surveillance.

Our general model, using parameters from the literature and animal distribution data available before the outbreak, closely matched the 2006–2007 influenza epidemic in Nigeria (see Fig 6). A hot-spot analysis indicated that LGAs with a similar number of positive model simulations were clustered together and that the locations of 2006–2007 epidemics were generally within statistically “hot” zones while “cold” zones contained fewer or no epidemic events. While our model results match the smoothed incidence risk of H5N1 during the outbreak from 2005–2008 compiled by Henning et al. [14], as well as the risk of Nigerian states calculated by Pelletier et al. [50], we show slightly less risk associated with northern Nigeria. This may be due the fact that the outbreaks were first concentrated in the northern zones of Nigeria during the first phase of the epidemic [21] and thus, was likely an initial introduction site. Pelletier et al. [50] demonstrated that location of the introduction of H5N1 into Nigeria greatly impacts the spread of a disease outbreak. As Pelletier et al. [50] point out with H5N1 in Nigeria; transmission is a stochastic process and not all introductions lead to major epidemics. This comparison of our theoretical simulation model with an actual outbreak is an important validation step for the model, indicating that the model captures enough of the variation and important parameters in the real system to be useful for designing surveillance.

The risk factors (in terms of final epidemic size) that we identified include the time before culling of birds occurs and the geographic location where an epidemic starts. We also note that the number (or fraction of total population) of birds that are infected before officials are alerted about the infection and the response begins can impact the size of the epidemic. This follows the established ideology regarding the importance of rapid detection of infectious diseases [51].

Recent highly pathogenic avian influenzas have been shown to travel large distances without leaving a trail of dead birds. While the spread of avian influenza across regions has been shown to be inevitable, detection of specific influenza in poultry flocks and resulting culling of the birds, has also been shown be highly effective [43]. The direct costs of culling poultry flocks is significant, and there are myriad other economic consequences for highly pathogenic avian influenza spread into regions, as documented in the H5N1 influenza epidemic in Nigeria [44].

Our analysis shows that reducing short range transportation in poultry can decrease the spread of the epidemic. This observations is similar to the results of [52], who found that local spread was the predominant transmission mechanism for the 2009 Vietnam H5N1 influenza outbreak. Our conclusions also support the work of [53] that highlight the importance of trade and proximity between poultry farms in the epidemiology of H5N1 and the role of biosecurity in disease prevention.

This analysis demonstrated that the epidemic start location makes an immense difference in the extent and severity of infections and propagation of the infectious disease. However, this analysis also demonstrated that regardless of where the infection begins, certain LGAs always have high potential for infection and hence these LGAs are good candidates for surveillance. Alternatively, surveillance can be focused outside of these areas with the intent of keeping the disease out of these important areas of disease propagation and transmission.

## Conclusion

Early detection of zoonotic disease outbreaks through effective biosurveillance is critical for quickly establishing disease mitigation measures that can extinguish an epidemic. Zoonotic infectious diseases have the additional challenge of the lack of incentives for reporting animal infections due to the extensive and uncertain economic consequences. We show that epidemiological modeling can be used in biosurveillance planning prior to an outbreak occurring to assist in highlighting regions of risk for a specific infectious disease. Surveillance is costly and time-dependent, although planning can be completed prior to an epidemic to assist in the effectiveness of detecting a disease and minimizing it effects. Using the full range of potential disease characteristics of highly pathogenic H5N1 influenza and different mitigation scenarios in Nigeria, we demonstrate that epidemiological models can be utilized far in advance of an outbreak to plan a biosurveillance strategy for a disease and a country.

## References

[1] Subbarao K, Klimov A, Katz J, Regnery H, Lim W, et al (1998) Characterization of an avian influenza A (H5N1) virus isolated from a child with a fatal respiratory illness. Science 279: 393–396. 9430591 10.1126/science.279.5349.393

[2] Paul M, Baritaux V, Wongnarkpet S, Poolkhet C, Thanapongtharm W, et al (2013) Practices associated with highly pathogenic avian influenza spread in traditional poultry marketing chains: social and economic perspectives. Acta Tropica 126: 43–53. 10.1016/j.actatropica.2013.01.008 23337390

[3] Shi J, Deng G, Liu P, Zhou J, Guan L, et al (2013) Isolation and characterization of H7N9 viruses from live poultry markets—Implication of the source of current H7N9 infection in humans. Chinese Science Bulletin: 1–7.

[4] Pritchett J, Thilmany D, Johnson K (2005) Animal disease economic impacts: A survey of the literature and research approaches. Internat Food Agribusiness Manage Rev 8: 23–45.

[5] Fasina FO, Sirdar MM, Bisschop SPR (2008) The financial cost implications of the highly pathogenic notifiable avian influenza H5N1 in Nigeria. Onderstepoort Journal of Veterinary Research 75: 39–46. 18575062 10.4102/ojvr.v75i1.86

[6] McNabb SJN (2010) Comprehensive effective and efficient global public health surveillance. Bmc Public Health 10. 10.1186/1471-2458-10-S1-S3 PMC3005575 21143825

[7] OIE (2009) Launch of global early warning system for animal diseases transmissible to humans. Geneva: OIE.

[8] GAO (2011) Nonfederal Capabilities Should Be Considered in Creating a National Biosurveillance Strategy. Washington, D.C.: United States Government Accountability Office.

[9] Warns-Petit E, Artois M, Calavas D (2009) Wildlife biosurveillance. Bulletin De L Academie Veterinaire De France 162: 205–213.

[10] Joannis T, Lombin LH, De Benedictis P, Cattoli G, Capua I (2006) Confirmation of H5N1 avian influenza in Africa. Veterinary Record 158: 309–310. 16517825 10.1136/vr.158.9.309-b

[11] Briand S (2010) Overview of human cases of avian influenza since 1997. Influenza and Other Respiratory Viruses 4: 31–31.

[12] Okoye J, Eze D, Krueger WS, Heil GL, Friary JA, et al (2013) Serologic evidence of avian influenza virus infections among Nigerian agricultural workers. Journal of Medical Virology 85: 670–676. 10.1002/jmv.23520 23400898

[13] Ortiz JR, Katz MA, Mahmoud MN, Ahmed S, Bawa SI, et al (2007) Lack of evidence of avian-to-human transmission of avian influenza A (H5N1) virus among poultry workers, Kano, Nigeria, 2006. Journal of Infectious Diseases 196: 1685–1691. 18008254 10.1086/522158

[14] Henning J, Bett B, Okike I, Abdu P, Perry B (2012) Incidence of highly pathogenic avian influenza H5N1 in Nigeria, 2005–2008. Transboundary and Emerging Diseases: no-no. 10.1111/j.1865-1682.2012.01331.x 22530694

[15] Fasina FO, Rivas AL, Bisschop SPR, Stegeman AJ, Hernandez JA (2011) Identification of risk factors associated with highly pathogenic avian influenza H5N1 virus infection in poultry farms, in Nigeria during the epidemic of 2006–2007. Preventive Veterinary Medicine 98: 204–208. 10.1016/j.prevetmed.2010.11.007 21146235

[16] Bavinck V, Bouma A, van Boven M, Bos MEH, Stassen E, et al (2009) The role of backyard poultry flocks in the epidemic of highly pathogenic avian influenza virus (H7N7) in the Netherlands in 2003. Preventive Veterinary Medicine 88: 247–254. 10.1016/j.prevetmed.2008.10.007 19178969

[17] Tiensin T, Nielen M, Vernooij H, Songserm T, Kalpravidh W, et al (2007) Transmission of the highly pathogenic avian influenza virus H5N1 within flocks during the 2004 epidemic in Thailand. Journal of Infectious Diseases 196: 1679–1684. 18008253 10.1086/522007

[18] Thompson PN, Sinclair M, Ganzevoort B (2008) Risk factors for seropositivity to H5 avian influenza virus in ostrich farms in the Western Cape Province, South Africa. Preventive Veterinary Medicine 86: 139–152. 10.1016/j.prevetmed.2008.03.011 18486977

[19] Nishiguchi A, Kobayashi S, Yamamoto T, Ouchi Y, Sugizaki T, et al (2007) Risk factors for the introduction of avian influenza virus into commercial layer chicken farms during the outbreaks caused by a low-pathogenic H5N2 virus in Japan in 2005. Zoonoses and Public Health 54: 337–343. 18035971 10.1111/j.1863-2378.2007.01074.x

[20] Rivas AL, Chowell G, Schwager SJ, Fasina FO, Hoogesteijn AL, et al (2010) Lessons from Nigeria: the role of roads in the geo-temporal progression of avian influenza (H5N1) virus. Epidemiology and Infection 138: 192–198. 10.1017/S0950268809990495 19653927

[21] Ekong PS, Ducheyne E, Carpenter TE, Owolodun OA, Oladokun AT, et al (2012) Spatio-temporal epidemiology of highly pathogenic avian influenza (H5N1) outbreaks in Nigeria, 2006–2008. Preventive Veterinary Medicine 103: 170–177. 10.1016/j.prevetmed.2011.10.001 22079423

[22] Bajardi P, Barrat A, Savini L, Colizza V (2012) Optimizing surveillance for livestock disease spreading through animal movements. Journal of the Royal Society Interface 9: 2814–2825. 22728387 10.1098/rsif.2012.0289 PMC3479905

[23] Manore C, McMahon B, Fair J, Hyman J, Brown M, et al (2011) Disease properties, geography, and mitigation strategies in a simulation spread of rinderpest across the United States. Veterinary Research 42: 55 10.1186/1297-9716-42-55 21435236 PMC3072946

[24] Wint W, Robinson T (2007) Gridded livestock of the world 2007. Food and Agricultural Organization of the Unted Nations. 20422554

[25] Lehner B, Doll P (2004) Development and validation of a global database of lakes, reservoirs and wetlands. Journal of Hydrology 296: 1–22.

[26] Adene DF, Oguntade AE (2008) The structure and importance of the commercial and village based poultry industry in Nigeria Rome, Italy: Food and Agricultural Organization of the United Nations.

[27] Cecchi G, Ilemobade A, Le Brun Y, Hogerwerf L, Slingenbergh J (2008) Agro-ecological features of the introduction and spread of the highly pathogenic avian influenza (HPAI) H5N1 in northern Nigeria. Geospatial Health 3: 7–16. 19021104 10.4081/gh.2008.227

[28] Tang B (1993) Orthogonal array-based latin hypercubes. Journal of the American Statistical Association 88: 1392–1397.

[29] Morris MD, Moore LM, McKay MD (2008) Using orthogonal arrays in the sensitivity analysis of computer models. Technometrics 50: 205–215.

[30] Fair JM, Powell DR, LeClaire RL, Moore LD, Wilson ML, et al (2012) Uncertainity in pandemic influenza. International Journal of Risk Assessment and Management.

[31] Zhong L, Zhao Q, Zhao K, Wang X, Zhao G, et al (2014) The antigenic drift molecular basis of the H5N1 influenza viruses in a novel branch of clade 2.3.4. Veterinary Microbiology 171: 23–30. 10.1016/j.vetmic.2014.02.033 24745625

[32] Johnson ME, Moore LM, Ylvisaker D (1990) Minimax and maximin distance designs. Journal of Statistical Planning and Inference 26: 131–148.

[33] McKay MD, Beckman RJ, Conover WJ (2000) A comparison of three methods for selecting values of input variables in the analysis of output from a computer code. Technometrics 42: 55–61.

[34] Wu C, Hamada MS (2000) Experiments: Planning, Analysis, and Parameter Design. New York, NY: John Wiley & Sons.

[35] Santner TJ, Williams BJ, Notz WI (2003) The Design and Analysis of Computer Experiments. New York, NY: Springer-Verlag

[36] McKay MD (1995) Evaluating prediction uncertainty. Los Alamos National Laboratory.

[37] McKay MD (1996) Variance-based methods for assessing uncertainty importance in HUREG-1150 analysis.

[38] Ord JK, Getis A (1995) Local spatial autocorrelation statistics: distributional issues and an application. Geographic Analysis 4: 286–306.

[39] Ducatez MF, Olinger CM, Owoade AA, Tarnagda Z, Tahita MC, et al (2007) Molecular and antigenic evolution and geographical spread of H5N1 highly pathogenic avian influenza viruses in western Africa. Journal of General Virology 88: 2297–2306. 17622635 10.1099/vir.0.82939-0

[40] Rich KM, Denwood MJ, Stott AW, Mellor DJ, Reid SWJ, et al (2013) Systems approaches to animal Disease surveillance and resource allocation: methodological frameworks for behavioral analysis. PLoS ONE 8: e82019 10.1371/journal.pone.0082019 24348922 PMC3857842

[41] Bian L (2013) Spatial approaches to modeling dispersion of communicable diseases—A Review. Transactions in Gis 17: 1–17.

[42] Maher SP, Kramer AM, Pulliam JT, Zokan MA, Bowden SE, et al (2012) Spread of white-nose syndrome on a network regulated by geography and climate. Nature Communications 3. 10.1038/ncomms2301 23250436

[43] Martin V, Chevalier V, Ceccato P, Anyamba A, De Simone L, et al (2008) The impact of climate change on the epidemiology and control of Rift Valley fever. Revue Scientifique Et Technique-Office International Des Epizooties 27: 413–426. 18819669

[44] Peterson AT (2006) Ecologic niche modeling and spatial patterns of disease transmission. Emerging Infectious Diseases 12: 1822–1826. 17326931 10.3201/eid1212.060373 PMC3291346

[45] Rivas AL, Fasina FO, Hoogesteyn AL, Konah S, Perkins DJ, et al (2012) Connecting network properties of rapidly disseminating epidemics. PloS One In Press. 10.1371/journal.pone.0039778 PMC3382573 22761900

[46] Nguyen AM, Noymer A (2013) Influenza Mortality in the United States, 2009 Pandemic: Burden, Timing and Age Distribution. Plos One 8. 10.1371/journal.pone.0064198 PMC3661470 23717567

[47] Van Kerkhove MD, Hirve S, Koukounari A, Mounts AW, Grp HNpSW (2013) Estimating age-specific cumulative incidence for the 2009 influenza pandemic: a meta-analysis of A(H1N1)pdm09 serological studies from 19 countries. Influenza and Other Respiratory Viruses 7: 872–886. 10.1111/irv.12074 23331969 PMC5781221

[48] Childs JE, Gordon ER (2009) Surveillance and control of zoonotic agents prior to disease detection in humans. Mount Sinai Journal of Medicine 76: 421–428. 10.1002/msj.20133 19787654

[49] Cannon RM (2005) Inspecting and monitoring on a restricted budget—where best to look? Preventive Veterinary Medicine 92: 163–174. 10.1016/j.prevetmed.2009.06.009 19640598

[50] Pelletier STK, Rorres C, Macko PC, Peters S, Smith G (2012) Models of highly pathogenic avian influenza epidemics in commercial poultry flocks in Nigeria and Ghana. Tropical Animal Health and Production 44: 1681–1687. 10.1007/s11250-012-0124-2 22476732 PMC3627490

[51] Morens DM, Folkers GK, Fauci AS (2004) The challenge of emerging and re-emerging infectious diseases. Nature 430: 242–249. 15241422 10.1038/nature02759 PMC7094993

[52] Minh PQ, Stevenson MA, Jewell C, French N, Schauer B (2011) Spatio-temporal analyses of highly pathogenic avian influenza H5N1 outbreaks in the Mekong River Delta, Vietnam, 2009. Spatial and Spatio-temporal Epidemiology 2: 49–57. 10.1016/j.sste.2010.11.001 22749548

[53] Metras R, Stevens KB, Abdu P, Okike I, Randolph T, et al (2013) Identification of potential risk factors associated with highly pathogenic avian influenza subtype H5N1 outbreak occurrence in Lagos and Kano States, Nigeria, during the 2006–2007 epidemics. Transboundary and Emerging Diseases 60: 87–96. 10.1111/j.1865-1682.2012.01322.x 22469078

